# Neuroanatomical and neuroimaging biomarkers of “MRI-defined vascular depression”

**DOI:** 10.1192/j.eurpsy.2025.731

**Published:** 2025-08-26

**Authors:** A. Starcevic, B. Vucinic, F. Starcevic, B. Starcevic, M. Stojanovic

**Affiliations:** 1 Anatomy; 2 University of Belgrade; 3 University Clinical Center of Belgrade, Belgrade; 4 Private practice Ortodent, Paracin, Serbia

## Abstract

**Introduction:**

“MRI-defined vascular depression” suggests that vascular lesions and small vessels disease induce depression by disruption of frontal–subcortical–limbic networks.Vascular depression is associated with disruption or cortico-striato-pallido-thalamo-cortical pathways or their modulating systems.

**Objectives:**

Based on clinical correlates and structural MRI findings we aim to evaluate causal relationship between specific brain changes and its related lesions in localization and number, through visual rating scales semi-automated and fully automated volumetric methods of specific software packages as part of deep machine learning.

**Methods:**

We have included 50 T2/FLAIR MRI brain scanned images, 30 patients (both genders) with late-onset vascular depression and 20 controls. In all subjects, T2/fluid-attenuated inversion recovery (FLAIR) sequences of the brain were collected during a single session using a 3 Tesla scanner (Siemens Skyra Medical Systems). FLAIR-white matter hyperintense lesions were identified and quantified using a local thresholding segmentation technique using specific software. FLAIR lesion volume and number was reported for the whole brain and for each hemisphere separately, without distinction between deep and periventricular.

**Results:**

There is statistical significance in total number and total regional volume of interest in brains of patients with late-onset vascular depression compared to controls (p<0.05). Median number of white matter hyperintensities was 14 per patient, and white matter hyperintensities median volume was 721 mm3. No difference was found between right and left hemisphere in terms of number (p>0.05) and volume (p>0.05) of white matter hyperintensities. Statistical significance was faound in volume and localisation of lesions in the brain (p<0.05).

**Image 1:**

**Figure:**
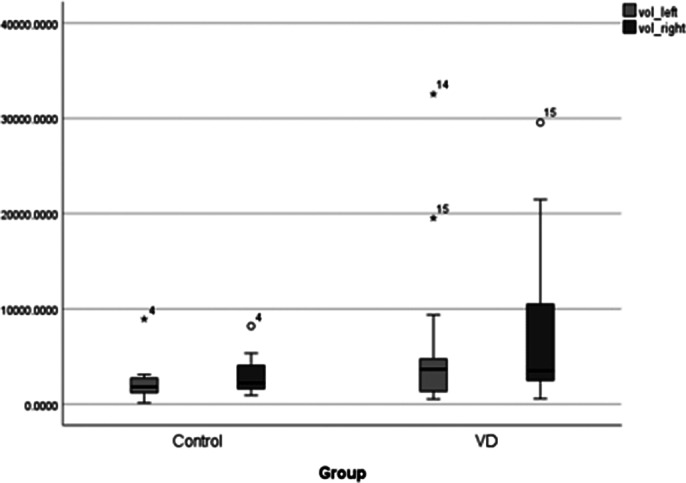

**Image 2:**

**Figure:**
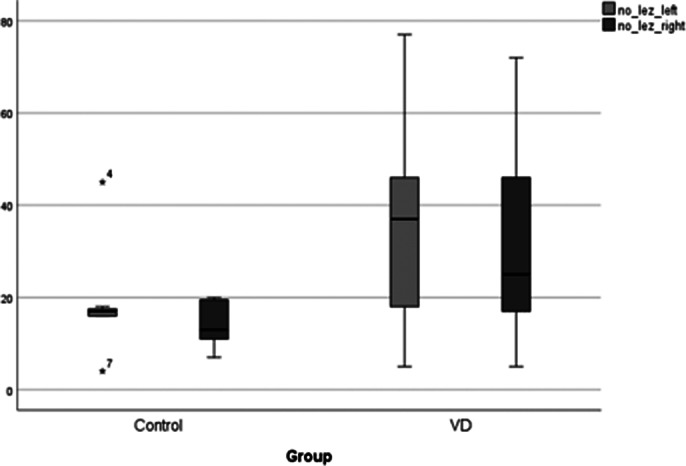

**Image 3:**

**Figure:**
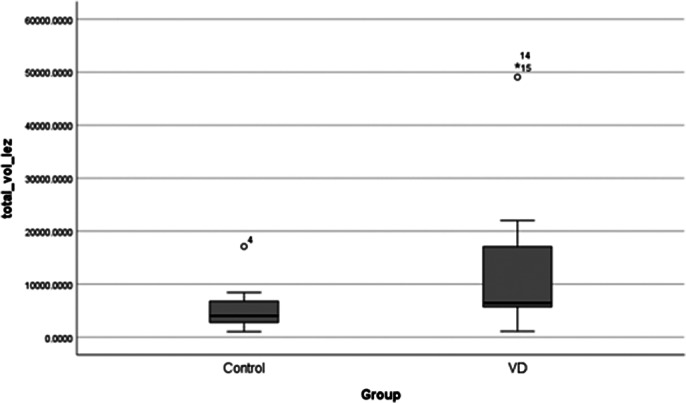

**Conclusions:**

Higher burden of white matter hyperintensities in patients with vascular depression could be associated with progression of clinical depressive symptomatology as well as with severity of brain damage.

**Disclosure of Interest:**

None Declared

